# Non-invasive liver fibrosis assessment and HCV treatment initiation within a systematic screening program in HIV/HCV coinfected patients

**DOI:** 10.1007/s00508-017-1231-x

**Published:** 2017-07-25

**Authors:** David Chromy, Philipp Schwabl, Theresa Bucsics, Bernhard Scheiner, Robert Strassl, Florian Mayer, Maximilian C. Aichelburg, Katharina Grabmeier-Pfistershammer, Michael Trauner, Markus Peck-Radosavljevic, Thomas Reiberger, Mattias Mandorfer

**Affiliations:** 1 Division of Gastroenterology and Hepatology, Department of Internal Medicine III, Medical University of Vienna, Währinger Gürtel 18–20, 1090 Vienna, Austria; 2 Vienna HIV & Liver Study Group, Medical University of Vienna, Vienna, Austria; 3 Department of Laboratory Medicine, Division of Clinical Virology, Medical University of Vienna, Vienna, Austria; 4 Division of Immunology, Allergy and Infectious Diseases, Department of Dermatology, Medical University of Vienna, Vienna, Austria

**Keywords:** HIV, Hepatitis C, Liver Cirrhosis, Elasticity Imaging Techniques

## Abstract

**Background and aim:**

Hepatitis C virus (HCV) therapy should be considered without delay in all patients with significant (SIGFIB) or advanced liver fibrosis (ADVFIB). We aimed to investigate the rates of treatment initiation with interferon-free regimens within a screening program for SIGFIB/ADVFIB in human immunodeficiency virus/HCV coinfected patients (HIV/HCV).

**Methods:**

The FIB-4 was calculated in all HIV/HCV from 2014–2016. HIV/HCV were counselled by the HIV clinic and referred to the Division of Gastroenterology and Hepatology for transient elastography (TE) and evaluation for HCV therapy. Patients were stratified by FIB-4 of </≥1.45 (established cut-off for ruling out ADVFIB) and SIGFIB/ADVFIB were defined by liver stiffness >7.1 kPa/>9.5 kPa, respectively.

**Results:**

Among 1348 HIV+ patients, 16% (210/1348) had detectable HCV-RNA. One hundred HIV/HCV had a FIB-4 ≥1.45. Among these, 57% (57/100) underwent TE. The majority of these patients had SIGFIB (75%; 43/57) or ADVFIB (37%; 21/57), however, interferon-free treatment was initiated in only 56% (24/43).

In addition, fifty-two percent (57/110) of HIV/HCV with FIB-4 <1.45 underwent TE. Interestingly, 40% (23/57) and 18% (10/57) of these patients showed SIGFIB or even ADVFIB, respectively, and 78% (18/23) finally received interferon-free treatment. Overall, only 20% (42/210) of HIV/HCV received interferon-free treatment.

**Conclusion:**

FIB-4 was not useful for ruling out SIGFIB/ADVFIB in our cohort of HIV/HCV. Treatment was initiated only in a small proportion (20%) of HIV/HCV during the first 2 years of interferon-free treatment availability, although the observed proportion of patients with SIGFIB (assessed by TE) was considerably higher (58%). Thus, it requires the ongoing combined efforts of both HIV and HCV specialists to increase treatment uptake rates in this special population.

**Electronic supplementary material:**

The online version of this article (doi: 10.1007/s00508-017-1231-x) contains supplementary material, which is available to authorized users.

**Fig. 1:**
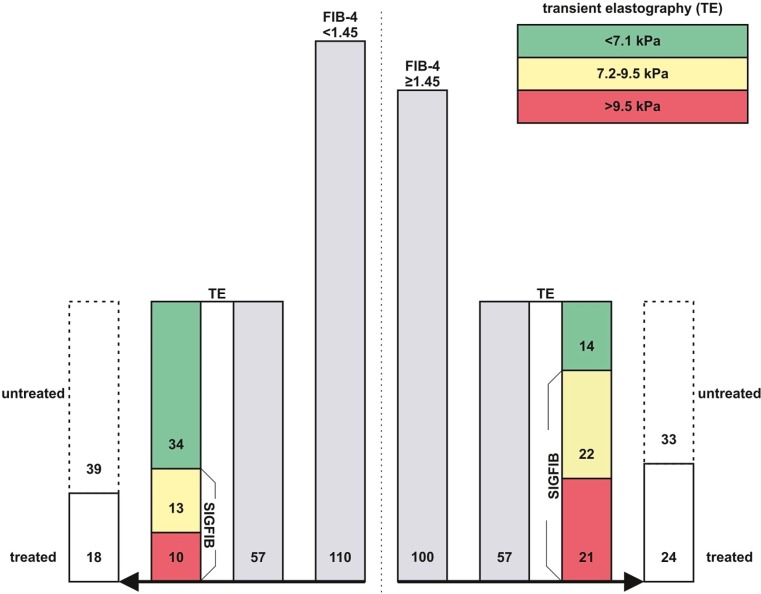
Proportions of patients undergoing liver fibrosis assessment by transient elastography (TE) and HCV treatment stratified by FIB-4 index. Of the HIV/HCV coinfected patients 100 had a FIB-4 ≥1.45 (right panel). Among these, 57% (57/100) underwent TE. The majority of these patients had significant (SIGFIB, 75%; 43/57) or advanced liver fibrosis (ADVFIB, 37%; 21/57), however, interferon-free treatment was initiated in only 56% (24/43). In addition, 52% (57/110) of HIV/HCV coinfected patients with FIB-4 <1.45 underwent TE (left panel). Interestingly, 40% (23/57) and 18% (10/57) of these patients showed SIGFIB or even ADVFIB, respectively, and 78% (18/23) finally received interferon-free treatment

## Introduction

Worldwide, approximately 80 million people suffer from chronic hepatitis C (CHC) [1]. In low prevalence areas like Central and Western Europe the main route of transmission is intravenous drug abuse (IVDA) [2], which is also associated with a high risk of human immunodeficiency virus/hepatitis C virus (HIV/HCV) coinfection [3, 4]. In addition, there is an ongoing epidemic of acute hepatitis C among HIV-positive men who have sex with men (MSM) [5]. With 85% of acute HCV infections in HIV-positive patients resulting in CHC [6], the prevalence of CHC among HIV-positive persons in Western Europe and the USA is estimated to be as high as 25–30% [2]. An HIV/HCV coinfection is associated with accelerated progression towards advanced liver disease as compared to HCV monoinfections [7] and thus, making HCV-associated liver disease a major contributor to morbidity and mortality in HIV-positive patients [8]; however, novel interferon-free (IFN-free) directly acting antiviral (DAA) regimens have largely improved sustained virological response (SVR) rates in HIV/HCV coinfection: following the promising results of the initial studies using sofosbuvir (SOF) and ribavirin (RBV) [9], several studies investigating second generation DAA combination regimens, such as SOF/daclatasvir (DCV) [10], SOF/ledipasvir (LDV) [11], ombitasvir/ritonavir-boosted paritaprevir ± dasabuvir (2D/3D) [12], and grazoprevir (GZV)/elbasvir (EBV) [13, 14] reported excellent SVR rates exceeding 95%. According to the European Association for the Study of the Liver (EASL) recommendations [15], the treatment indications for HIV/HCV coinfected patients are identical to HCV monoinfections. While all patients with CHC should be considered for antiviral therapy, treatment should not be delayed in patients with significant fibrosis (SIGFIB, i. e. METAVIR ≥ fibrosis stage 2). Nevertheless, in many countries including Austria reimbursement of IFN-free treatment is commonly restricted to patients with SIGFIB or patients infected with a specific HCV genotype due to its substantial impact on the health insurance budget [16]. Thus, assessment of the severity of liver fibrosis is crucial for treatment initiation. The most widely used non-invasive method for the assessment of liver fibrosis is transient elastography (TE), which is accepted as a surrogate of liver fibrosis and substitute for invasive liver biopsy by the Austrian health insurances [17].

The FIB-4, an index based on simple laboratory parameters, e.g. age, aspartate aminotransferase (AST), platelet count (PLT), alanine aminotransferase (ALT) has been developed to rule out (FIB-4 <1.45: negative predictive value, NPV: 90%) or include (FIB-4 >3.25: positive predictive value, PPV: 65%) advanced fibrosis (ADVFIB, i. e. METAVIR ≥F3) in HIV/HCV coinfected patients [18] and has been extensively validated against liver biopsies [19]. Importantly, FIB-4 is readily available and can thus be easily applied in cohorts of HIV/HCV coinfected to identify patients with increased risk for SIGFIB or ADVFIB and in whom treatment should not be delayed.

Within this systematic screening project, HIV/HCV coinfected patients were counselled by the HIV clinic and referred to the Division of Gastroenterology and Hepatology for TE and evaluation for HCV therapy.

The study aimed to explore whether FIB-4 index is an accurate tool to identify patients who are at considerable risk for SIGFIB or ADVFIB, and thus, might have an urgent need for antiviral therapy. Moreover, the proportions of patients with DDIs between DAA-based regimens and antiretroviral therapy (ART) as well as the rates of treatment uptake in a cohort of HIV/HCV coinfected patients were assessed.

## Methods

### Study design and population

Within this systematic screening project, HIV/HCV coinfected patients were counselled by the HIV clinic and referred to the Division of Gastroenterology and Hepatology for TE and evaluation for HCV therapy between 2014 and 2016. All HIV-positive patients with chronic hepatitis C were included in this retrospective analysis of the screening program.

### Assessed parameters

Epidemiological characteristics were assessed from patient medical history. The HCV genotype was determined using the VERSANT® HCV Genotype 2.0 Assay Line Probe Assay (LiPA, Siemens Healthcare Diagnostics, Tarrytown, NY) and HCV-RNA was assessed using the Abbott RealTime HCV assay (Abbott Molecular, Des Plaines, IL) with a lower limit of quantification and detection of 12 IU ml^−1^.

### FIB-4 and liver stiffness measurement

Measurement of liver stiffness was performed by TE (Fibroscan®, Echosens, Paris, France), as previously described [20, 21]. The FIB-4 was calculated as age (years) × AST (U/l) × PLT (10^9^/l) × ALT (U/l)^1/2^)^−1^ [18]:

The SIGFIB and ADVFIB were defined by liver stiffness values >7.1 and >9.5 kPa, respectively [22]. Patients were stratified according to FIB-4 of 1.45, a previously established cut-off for ruling out ADVFIB [23].

### Statistical analysis

Statistical analyses were performed using IBM SPSS Statistics 23 (SPSS, Armonk, NY, USA). Initially, normal distribution of continuous variables was tested by applying the Kolmogorov-Smirnov test to both the entire study population and to each individual subgroup. Continuous variables were reported as mean ± standard deviation or median (interquartile range), while categorical variables were reported as number of patients with/without (proportion of patients with) the certain characteristics.

Student’s t‑test was used for group comparisons of continuous variables when applicable. Otherwise, the Mann-Whitney U-test was applied. Group comparisons of categorical variables were performed using the χ^2^-test and Fisher’s exact test. A *P* value of ≤0.05 was considered statistically significant.

## Results

### Patient characteristics

Among the 1348 HIV-positive patients counselled by the HIV clinic, 33% (439/1348) were HCV-antibody positive and 16% (210/1348) of patients had detectable HCV-RNA. The majority of HIV/HCV coinfected patients were male (65%) and the median age was 37.9 (SD±16.61) years. The main route of transmission was IVDA (75%) followed by heterosexual intercourse (14%) and 5% of patients were MSM. More than half of the patients were coinfected with HCV genotype 1 (57%), while HCV genotypes 2, 3, and 4 were observed in 1, 10, and 32% of patients, respectively. Among HCV genotype 1 patients the subtype 1a (76%) was more common than subtype 1b (24%). The vast majority of the patients were on ART (91%), with 63% of HIV/HCV-coinfected patients having suppressed HIV-RNA (<50 copies ml^−1^). The vast majority received at least one nucleoside reverse transcriptase inhibitor (N(t)RTI, 93%), followed by HIV protease inhibitor (PI, 57%) and non-nucleoside reverse transcriptase inhibitor (NNRTI, 18%) treatment. Of the patients one quarter (25%) received either an integrase inhibitor or an entry inhibitor (II/EI) as part of their ART.

### Comparison of patients with and without information on TE

**Table 1:** Comparison of HIV/HCV-coinfected patients who underwent liver fibrosis assessment by transient elastography (TE) vs. patients who did not

Patient characteristics	All patients (*n* = 210)	Underwent TE (*n* = 114)	Without TE (*n* = 96)	*P*-value
**Epidemiological characteristics**				
*Sex*				
Male	65% (136/210)	68% (77/114)	61% (59/96)	0.358
Female	35% (74/210)	32% (37/114)	39% (37/96)	
*Age*	39.1 ± 10.8	41.5 ± 11.0	36.3 ± 10.0	<0.001
*Transmission*				
MSM	5% (10/210)	7% (8/114)	2% (2/96)	0.233
IVDU	75% (158/210)	71% (81/114)	80% (77/96)	
Heterosexual	14% (30/210)	17% (19/114)	12% (11/96)	
Others	6% (12/210)	5% (6/114)	6% (6/96)	
**Laboratory parameters**				
Hemoglobin (g dl^−1^)	13.8 (2.55)	13.8 (2.25)	13.6 (2.90)	0.066
Platelet count (10^9^ l^−1^)	196 (96.3)	195 (94)	199 (102)	0.731
White blood cell count (10^9^ l^−1^)	6.42 (3.35)	6.45 (3.38)	6.28 (3.36)	0.900
Prothrombin time (%)	95.4 ± 25.4	97.3 ± 25.4	92.9 ± 25.1	0.249
Albumin (g dl^−1^)	42.7 (6)	43.2 (5.8)	42.0 (7.2)	<0.001
Creatinine (mg dl^−1^)	0.82 (0.26)	0.84 (0.24)	0.81 (0.30)	0.189
Bilirubin (mg dl^−1^)	0.50 (0.4)	0.50 (0.46)	0.49 (0.39)	0.524
AST (U l^−1^)	44.0 (31)	43.5 (27.3)	47.0 (35.5)	0.474
ALT (U l^−1^)	40.5 (42.8)	41.0 (28.5)	38.5 (58.0)	0.847
GGT (U l^−1^)	76.0 (88.0)	76.0 (86.0)	70.5 (96.3)	0.973
**HIV infection parameters**				
*CD4+ T‑lymphocyte count (cells μl* ^*−1*^ *)*	455 (368)	511 (414)	352 (380)	0.001
*HIV-RNA <50 copies ml* ^*−1*^	63% (130/207)	74% (84/113)	49% (46/94)	<0.001
*HIV-RNA <400 copies ml* ^*−1*^	76% (157/207)	89% (100/113)	61% (57/94)	<0.001
*cART*	91% (190/210)	95% (108/114)	85% (82/96)	0.022
PI	57% (108/190)	44% (48/108)	73% (60/82)	<0.001
N(t)RTI	93% (177/190)	94% (102/108)	92% (75/82)	0.420
NNRTI	18% (35/190)	21% (23/108)	15% (12/82)	0.241
II/EI	25% (47/190)	35% (38/108)	11% (9/82)	<0.001
**HCV infection parameters**				
*HCV-RNA (log IU ml* ^*−1*^ *)*	5.93 (1.40)	6.00 (1.07)	5.75 (2.02)	0.214
*HCV genotype*	87% (183/210)	94% (107/114)	79% (76/96)	–
1	57% (104/183)	56% (60/107)	58% (44/76)	0.123
2	1% (3/183)	0% (0/107)	4% (3/76)	
3	32% (58/183)	32% (34/107)	32% (24/76)	
4	10% (18/183)	12% (13/107)	6% (5/76)	
*Liver stiffness*				
F0/F1 (<7.1 kPa)	–	42% (48/114)	–	–
F2 (≥7.1 and <9.5 kPa)	–	31% (35/114)	–	–
F3 (≥9.5 and <12.5 kPa)	–	7% (8/114)	–	–
F4 (≥12.5 kPa)	–	20% (23/114)	–	–
Significant liver fibrosis (≥7.1 kPa)	–	58% (66/114)	–	–
Advanced liver fibrosis (≥9.5 kPa)	–	27% (31/114)	–	–
FIB-4 <1.45	52% (110/210)	50% (57/114)	55% (53/96)	0.452
FIB-4 ≥1.45	48% (100/210)	50% (57/114)	45% (43/96)	

*ALT* alanine transaminase, *AST* aspartate transaminase, *cART* combined antiretroviral therapy, *EI* entry inhibitors, *GGT* gamma-glutamyl transpeptidase, *GT* genotype, *HCV* hepatitis C virus, *HIV* human immunodeficiency virus, *II* integrase inhibitors, *IVDU* intravenous drug abuse, *MSM* men who have sex with men, *NNRTI* non-nucleoside reverse transcriptase inhibitors, *N(t)RTIs* nucleos(t)idic reverse transcriptase inhibitors, *PI* protease inhibitor, *TE* transient elastography

Of the HIV/HCV coinfected patients 114 (54%) underwent TE, while 96 (46%) patients did not (Table 1). There were a statistically significant differences in age (41.5 ± 11.0 vs. 36.3 ± 10.0 years; *P* < 0.001) and patients who underwent TE showed a better immune status, i. e. a higher CD4+ T‑lymphocyte count (511 (414) vs. 352 (380) cells μl^−1^; *P* = 0.001), proportion of patients with HIV-RNA <50 copies ml^−1^ (74% (84/113) vs. 49% (46/94); *P* < 0.001) and HIV-RNA <400 copies ml^−1^ (89% (100/113) vs. 61% (57/94); *P* < 0.001). Moreover, TE patients were more likely to receive an II/EI (35% (38/108) vs. 11% (9/82); *P* < 0.001). The opposite applied for HIV PI (44% (48/108) vs. 73% (60/82); *P* < 0.001). The proportion of patients with FIB-4 index ≥1.45 was comparable between patients with (50% (57/114)) and without (45% (43/96)) information on TE (*P* = 0.452).

### Comparison of patients with FIB-4 index <1.45 and FIB-4 ≥1.45

Of the patients 110 had a FIB-4 index <1.45 and 100 patients presented with a FIB-4 index ≥1.45 (Supplementary Table 1; Fig. 1). Besides statistically significant differences in the variables included in the FIB-4 index (e.g. age, platelet count, AST and ALT), we observed a statistically significantly higher CD4+ T‑lymphocyte count (514 (426.8) vs. 353 (362) cells μl^−1^; *P* = 0.002) among patients with a FIB-4 index <1.45. Moreover, bilirubin was lower among patients with a FIB-4 index <1.45 (0.43 (0.34) vs. 0.54 (0.46) mg dl^−1^; *P* < 0.001). The proportion of patients who underwent TE was comparable between patients with a FIB-4 index <1.45 (52% (57/110)) and ≥1.45 (57% (57/100); *P* = 0.452).

### Comparison of patients with FIB-4 index <1.45 and FIB-4 ≥1.45 who underwent TE

**Table 2:** Comparison of HIV/HCV-coinfected patients with FIB-4 <1.45 and ≥1.45 who underwent transient elastography

Patient characteristics	All patients (*n* = 114)	FIB-4 <1.45 (*n* = 57)	FIB-4 ≥1.45 (*n* = 57)	*P*-value
**Epidemiological characteristics**				
*Sex*				
Male	68% (77/114)	72% (41/57)	63% (36/57)	0.317
Female	33% (37/114)	28% (16/57)	37% (21/57)	
*Age*	43.5 (17.7)	34.2 (16.1)	49.4 (11.0)	<0.001
*Transmission*				
MSM	7% (8/114)	5% (3/57)	9% (5/57)	0.648
IVDU	71% (81/114)	72% (41/57)	70% (40/57)	
Heterosexual	17% (19/114)	19% (11/57)	14% (8/57)	
Others	5% (6/114)	4% (2/57)	7% (4/57)	
**Laboratory parameters**				
Hemoglobin (g dl^−1^)	13.8 (2.85)	13.9 (2.32)	13.8 (2.62)	0.375
Platelet count (G l^−1^)	200 ± 74.62	241 ± 63.8	158 ± 60.7	<0.001
White blood cell count (G l^−1^)	6.54 ± 2.67	7.28 ± 2.11	5.79 ± 2.97	0.003
Prothrombin time (%)	93.0 (33.5)	96.0 (37.5)	91.5 (39.3)	0.167
Albumin (g dl^−1^)	42.9 ± 4.66	43.5 ± 4.41	42.3 ± 4.86	0.166
Creatinine (mg dl^−1^)	0.84 (0.24)	0.80 (0.24)	0.88 (0.23)	0.205
Bilirubin (mg dl^−1^)	0.50 (0.46)	0.43 (0.34)	0.53 (0.46)	0.011
AST (U l^−1^)	43.5 (27.3)	37.0 (21.0)	51.0 (31.0)	<0.001
ALT (U l^−1^)	41.0 (28.5)	40.0 (22.5)	44.0 (39.5)	0.451
GGT (U l^−1^)	76.0 (86.0)	62.5 (75.8)	90.0 (80.0)	0.002
**HIV infection parameters**				
*CD4+ T‑lymphocyte count (cells μl* ^*−1*^ *)*	523 ± 272	628 ± 279	421 ± 224	<0.001
*HIV-RNA <50 copies ml* ^*−1*^	74% (84/113)	75% (42/56)	74% (42/57)	0.873
*HIV-RNA <400 copies ml* ^*−1*^	89% (100/113)	86% (48/56)	91% (78/57)	0.358
*cART*	95% (108/114)	93% (53/57)	97% (55/57)	0.679
PI	44% (48/108)	45% (24/53)	44% (24/55)	0.863
N(t)RTI	94% (102/108)	96% (51/53)	93% (51/55)	0.679
NNRTI	21% (23/108)	21% (11/53)	22% (12/55)	0.893
II/EI	35% (38/108)	36% (19/53)	35% (19/55)	0.887
**HCV infection parameters**				
*HCV-RNA (log IU ml* ^*−1*^ *)*	6.00 (1.07)	5.80 (1.23)	6.24 (0.99)	0.72
*HCV genotype*	94% (107/114)	93% (53/57)	95% (54/57)	–
1	56% (60/107)	58% (31/53)	54% (29/54)	0.145
2	0% (0/107)	0% (0/53)	0% (0/54)	
3	32% (34/107)	25% (13/53)	39% (21/54)	
4	12% (13/107)	17% (9/53)	7% (4/54)	
*Liver stiffness*				
F0/F1 (<7.1 kPa)	42% (48/114)	60% (34/57)	25% (14/57)	<0.001
F2 (≥7.1 and <9.5 kPa)	31% (35/114)	23% (13/57)	39% (22/57)	0.068
F3 (≥9.5 and <12.5 kPa)	7% (8/114)	7% (4/57)	7% (4/57)	1
F4 (≥12.5 kPa)	20% (23/114)	11% (6/57)	30% (17/57)	0.010
Significant liver fibrosis (≥7.1 kPa)	58% (66/114)	40% (23/57)	75% (43/57)	<0.001
Advanced liver fibrosis (≥9.5 kPa)	27% (31/114)	18% (10/57)	37% (21/57)	0.021

*ALT* alanine transaminase, *AST* aspartate transaminase, *cART* combined antiretroviral therapy, *EI* entry inhibitors, *GGT* gamma-glutamyl transpeptidase, *GT* genotype, *HCV* hepatitis C virus, *HIV* human immunodeficiency virus, *II* integrase inhibitors, *IVDU* intravenous drug abuse, *MSM* men who have sex with men, *NNRTI* non-nucleoside reverse-transcriptase inhibitors, *N(t)RTIs* nucleos(t)idic reverse transcriptase inhibitors, *PI* protease inhibitor

A total of 57 patients (52%) with a FIB-4 index <1.45 as well as 57 patients (50%) with a FIB-4 index ≥1.45 were evaluated by TE (Table 2; Fig. 1). Of the patients with a FIB-4 index <1.45, 40% (23/57) presented with a SIGFIB, and 18% (10/57) even showed ADVFIB. Among patients FIB-4 index ≥1.45, SIGFIB was observed in 86% (43/57; *P* = 0.001 when compared to FIB-4 index <1.45) and ADVFIB in 37% (21/57; *P* = 0.001 when compared to FIB-4 index <1.45).

### Treatment initiation

**Table 3:** Comparison of HIV/HCV coinfected patients who underwent transient elastography in whom HCV treatment was initiated vs. patients who did not undergo HCV treatment

Patient characteristics	All patients (*n* = 114)	No treatment (*n* = 72)	Treatment (*n* = 42)	*P*-value
**Epidemiological characteristics**				
*Sex*				
Male	68% (77/114)	75% (54/72)	55% (23/42)	0.026
Female	33% (37/114)	25% (18/72)	45% (19/42)	
*Age*	43.5 (17.7)	41.0 (16.3)	46.75 (16.9)	0.078
*Transmission*				
MSM	7% (8/114)	6% (4/72)	10% (4/42)	0.468
IVDU	71% (81/114)	74% (53/72)	67% (28/42)	
Heterosexual	17% (19/114)	14% (10/72)	21% (9/42)	
Others	5% (6/114)	7% (5/72)	2% (1/42)	
**Laboratory parameters**				
Hemoglobin (g dl^−1^)	13.8 (2.85)	13.9 (2.18)	13.6 (3.17)	0.106
Platelet count (G l^−1^)	200 ± 74.6	206 ± 66	189 ± 87.2	0.232
White blood cell count (G l^−1^)	6.54 ± 2.67	6.76 ± 2.67	6.15 ± 2.66	0.238
Prothrombin time (%)	93 (33.5)	89 (32.0)	98 (43.5)	0.589
Albumin (g dl^−1^)	42.91 ± 4.66	43.35 ± 4.99	42.14 ± 3.95	0.184
Creatinine (mg dl^−1^)	0.84 (0.24)	0.81 (0.18)	0.90 (0.35)	0.102
Bilirubin (mg dl^−1^)	0.50 (0.46)	0.50 (0.33)	0.52 (0.58)	0.920
AST (U l^−1^)	43.5 (27.3)	47.0 (33.0)	39.5 (25.3)	0.203
ALT (U l^−1^)	41.0 (28.5)	45.0 (36.8)	37.5 (24.8)	0.102
GGT (U l^−1^)	76.0 (86.0)	77.5 (86.3)	72.0 (83.0)	0.960
**HIV infection parameters**				
*CD4+ T‑lymphocyte count (cells μl* ^*−1*^ *)*	522.53 ± 272.21	514.76 ± 256.03	535.98 ± 301.02	0.693
*HIV-RNA <50 copies ml* ^*−1*^	74% (84/113)	72% (51/71)	79% (33/42)	0.428
*HIV-RNA <400 copies ml* ^*−1*^	89% (100/113)	86% (61/71)	93% (39/42)	0.264
*cART*	95% (108/114)	92% (66/72)	100% (42/42)	0.084
PI	44% (48/108)	53% (35/66)	31% (13/42)	0.024
N(t)RTI	94% (102/108)	96% (63/66)	93% (39/42)	0.676
NNRTI	21% (23/108)	23% (15/66)	19% (8/42)	0.649
II/EI	35% (38/108)	23% (15/66)	55% (23/42)	0.001
**HCV infection parameters**				
*HCV-RNA (log IU ml* ^*−1*^ *)*	6.0 (1.07)	5.97 (1.05)	6.11 (1.12)	0.645
*HCV genotype*	94% (107/114)	90% (65/72)	100% (42/42)	–
1	56% (60/107)	57% (37/65)	55% (23/42)	0.687
2	0% (0/107)	0% (0/65)	0% (0/42)	
3	32% (34/107)	29% (19/65)	36% (15/42)	
4	12% (13/107)	14% (9/65)	9% (4/42)	
*FIB-4*				
*<1.45*	50% (57/114)	54% (39/72)	43% (18/42)	0.244
*≥1.45*	50% (57/114)	46% (33/72)	57% (24/42)	0.244
*Liver stiffness*				
F0/F1 (<7.1 kPa)	42% (48/114)	60% (43/72)	12% (5/42)	0.001
F2 (≥7.1 and <9.5 kPa)	31% (35/114)	24% (17/72)	43% (18/42)	0.032
F3 (≥9.5 and <12.5 kPa)	7% (8/114)	3% (2/72)	14% (6/42)	0.028
F4 (≥12.5 kPa)	20% (23/114)	14% (10/72)	31% (13/42)	0.029
Significant liver fibrosis (≥7.1 kPa)	58% (66/114)	40% (29/72)	88% (37/42)	0.001
Advanced liver fibrosis (≥9.5 kPa)	27% (31/114)	17% (12/72)	45% (19/42)	0.001

*ALT* alanine transaminase, *AST* aspartate transaminase, *cART* combined antiretroviral therapy, *EI* entry inhibitors, *GGT* gamma-glutamyl transpeptidase, *GT* genotype, *HCV* hepatitis C virus, *HIV* human immunodeficiency virus, *II* integrase inhibitors, *IVDU* intravenous drug abuse, *MSM* men who have sex with men, *NNRTI* non-nucleoside reverse transcriptase inhibitors, *N(t)RTIs* nucleos(t)idic reverse transcriptase inhibitors, *PI* protease inhibitor

Among the patients who underwent TE, treatment was initiated in 37% (42/114) (Table 3; Fig. 1). Although gender did not affect the probability of undergoing TE (Table 1), women were more likely to receive therapy. While 45% (19/42) of patients in whom treatment was initiated were female, 75% (54/72) of patients who were not treated were male (*P* = 0.026).

Unsurprisingly, the prevalence of SIGFIB (88% (37/42) vs. 40% (29/72); *P* = 0.001) and ADVFIB (45% (19/42) vs. 17% (12/72); *P* = 0.001) were higher in patients in whom treatment was initiated.

Overall, treatment was initiated in only 20% (42/210) of a total of 210 HIV-positive patients with CHC. The following interferon-free regimens were prescribed:

- Sofosbuvir (SOF)/ribavirin (RBV) – 5% (2/42)
- SOF/daclatasvir (DCV) – 55% (23/42)
- SOF/ledipasvir (LDV) – 29% (12/42)
- Ombitasvir/paritaprevir/dasabuvir (3D) ± RBV – 12% (5/42)

### Potential drug-drug interactions between directly acting antiviral agents

The majority of HIV/HCV coinfected patients received ART (91%). According to current data on drug-drug interactions (DDI) between DAA and ART [24], the combination of SOF/LDV or SOF/DCV would have been considered safe in all patients. A change in the ART regimen in patients with HCV genotypes 1 and 4 would have been necessary in 27% (31/113) and 68% (77/113) for the 2D/3D or the GPV/ELV regimens, respectively.

## Discussion

Since HIV positive persons are at substantial risk of being coinfected with HCV due to the shared routes of transmission [2], the European AIDS Clinical Society recommends screening for HCV infection in HIV-positive persons on an annual basis [25]. The proportion of viremic HIV/HCV coinfected patients among all 1348 HIV-positive patients counselled by our HIV clinic was 16% (210/1348). The assessment of severity of liver fibrosis in HIV/HCV coinfected patients should be performed in all patients to determine urge for further treatment [15]; however, 46% of HIV/HCV coinfected patients did not undergo TE, and thus, had no access to modern IFN-free regimens, since until recently reimbursement of IFN-free treatment was primarily restricted to patients with SIGFIB in Austria.

We observed a significant difference in HIV infection parameters such as CD4+ T‑lymphocyte count and HIV-RNA between patients who underwent TE compared to the ones who did not. According to previous studies [26, 27], immune status might be considered as a surrogate of adherence. Motivational barriers for ART treatment uptake and depression are considered as additional barriers to optimal adherence in HIV/HCV coinfected patients, when compared to HIV monoinfected patients [28]. Hence, the substantial proportion of HIV/HCV coinfected patients without liver fibrosis assessment using TE might reflect the proportion of patients with limited compliance. This assumption is also supported by the statistically significantly lower proportion of patients on modern HIV integrase inhibitors in this group, since at our HIV clinic, HIV PI-based regimens are preferred in patients with suboptimal adherence.

Simple non-invasive indices have good diagnostic accuracy when combined with TE [29]. The FIB-4 is a non-invasive index for predicting ADVFIB which has initially been developed based on a cohort of HIV/HCV coinfected patients [18] and has been extensively validated in this setting [30–32]. Since FIB-4 values were comparable between patients who underwent TE and patients who did not, we were able to assess whether the FIB-4 index allows identification of patients who are at risk for SIGFIB or ADVFIB, and thus, might have an urgent need for antiviral therapy.

A FIB-4 cut-off of 1.45 had a sensitivity of 68% (95% confidence interval, 95%CI: 50–82%) and negative predictive value (NPV) of 82% (95%CI: 70–90%) for ruling out ADVFIB, which is numerically lower than the sensitivity (70%) and NPV (90%) reported in a previous study by Sterling et al. [18]. Similarly, specificity and PPV for ADVFIB (FIB-4 cut-off of 3.25) were numerically lower (93% vs. 97% and 54% vs. 65%, respectively), when compared to the study by Sterling et al. [18].Moreover, we aimed to assess whether the FIB-4 index allows identification of patients who are at risk for SIGFIB. Therefore, we stratified patients into two groups using a FIB-4 cut-off of 1.45. Specificity and PPV for identifying patients with SIGFIB were substantially lower (vs. ADVFIB) with 57 and 37%, respectively. Importantly, FIB-4 <1.45 did not rule out SIGFIB or ADVFIB, since 40% of patients with a FIB-4 <1.45 had SIGFIB and 18% even had ADVFIB. Thus, using the previously established FIB-4 cut-off of 1.45, FIB-4 index is not useful for prescreening HIV/HCV-coinfected patients for SIGFIB or ADVFIB.

Our analysis revealed that treatment was initiated in only 20% of all HIV/HCV coinfected patients. Modern IFN-free regimens have shown excellent results in clinical trials with SVR rates exceeding 95% [10–14]. Although the extensive inclusion and exclusion criteria of clinical trials have raised concerns about the generalizability of these findings [33], real-life studies have shown encouraging results in unselected patients [34–36]. Thus, considering the excellent efficacy and safety of modern regimens, it is essential to improve treatment uptake rates to reduce the burden of HCV-related advanced liver disease [34]. Particularly people who inject drugs [2] need to be addressed by customized concepts. A higher knowledge of HCV is associated with increased willingness for HCV treatment [37] and thus a general lack of awareness of HCV is still a major concern [38]. Moser et al. [39] recently described a promising approach to address patients who are on opioid substitution therapy. In order to receive opioid substitution, a visit in a low-threshold drug treatment facility was mandatory on a daily basis, allowing HCV treatment to be coadministered. This approach substantially improved adherence [39]. Moreover, HCV treatment outcomes are not affected by opioids [40]. Thus, considering the recently extended access to IFN-free regimens, such programs might decrease the incidence of HCV among patients with IVDA [41].

Patients coinfected with HIV/HCV are no longer considered as difficult to treat population [5]; however, DDIs are a major concern when prescribing DAA-based regimens, especially, when regimens including a HCV PI such as the 2D/3D regimen or GPV and ELV are used [15]. Our findings suggest that combinations of SOF/LDV or SOF/DCV could be prescribed safely with any ART used in our cohort. In contrast, a change in the ART regimen would be necessary in 27% for the 2D/3D regimen and in 68% if GPV/ELV are prescribed. In these cases, ART treatment options would be limited to NRTIs and II/EIs [15]. With respect to the common use of HIV PIs as part of ART, physicians would have to deal with additional difficulties. Since PIs appear to have a higher resistance barrier than IIs, ART treatment history needs to be evaluated carefully to avoid virologic failure [42].

Although IFN-free regimens are now reimbursed for most HIV-positive patients with CHC regardless of the severity of liver fibrosis, considerable compliance issues remain. Thus, it is unclear whether extending the reimbursement of IFN-free treatment to patients without SIGFIB will lead to the anticipated increase in treatment uptake rates. Additional studies are needed to investigate the underlying factors hindering liver fibrosis assessment and treatment uptake in order to promote HCV elimination in this special population.

In conclusion, FIB-4 was not useful for ruling out ADVFIB in our cohort of HIV/HCV. Treatment was initiated only in a small proportion of HIV/HCV during the first 2 years of IFN-free treatment availability, although the observed proportion (20%) of patients with SIGFIB (assessed by TE) was considerably higher (58%). Thus, it requires the ongoing combined efforts of both HIV and HCV specialists to increase treatment uptake rates in this special population.

## Caption Electronic Supplementary Material

Supplementary Table 1 Comparison of all HIV/HCV coinfected patients with FIB-4 <1.45 and ≥1.45
