# Supplementary material for: Non-invasive liver fibrosis assessment and HCV treatment initiation within a systematic screening program in HIV/HCV coinfected patients
Source: Wien Klin Wochenschr. 2017 Jul 25;130(3):105–14. doi: 10.1007/s00508-017-1231-x (PMC5816107; doi:10.1007/s00508-017-1231-x)
Supplement: Supplementary file 1 — Supplementary Table 1 Comparison of all HIV/HCV coinfected patients with FIB-4 <1.45 and ≥1.45 [file 508_2017_1231_MOESM1_ESM.docx]

**Supplementary material**

| **Patient characteristics** | **All patients**  **(n=210)** | **FIB-4 <1.45**  **(n=110)** | **FIB-4 ≥1.45**  **(n=100)** | **P-value** |
| --- | --- | --- | --- | --- |
| Epidemiological characteristics |  |  |  |  |
| Sex |  |  |  |  |
| Male | 65% (136/210) | 63% (79/110) | 67% (62/100) | 0.517 |
| Female | 35% (74/210) | 37% (41/110) | 33% (33/100) |  |
| Age | 37.9 (16.61) | 32.65 (13.35) | 46.24 (15.01) | <0.001 |
| Transmission |  |  |  |  |
| MSM | 5% (10/210) | 5% (5/110) | 5% (5/100) | 0.561 |
| IVDU | 75% (158/210) | 74% (81/110) | 77% (77/100) |  |
| Heterosexual | 14% (30/210) | 17% (19/110) | 11% (11/100) |  |
| Others | 6%(12/210) | 5% (5/110) | 7% (7/100) |  |
| Laboratory parameters |  |  |  |  |
| Hemoglobin (g dl^-1^) | 13.8 (2.55) | 13.6 (2.85) | 13.85 (2.35) | 0.465 |
| Platelet count (G l^-1^) | 196 (96.3) | 228 (68.5) | 154 (87.8) | <0.001 |
| White blood cell count (G l^-1^) | 6.42 (3.35) | 7.02 (2.99) | 5.47 (3.28) | <0.001 |
| Prothrombin time (%) | 95.4 ±25.4 | 98.3 ±22.0 | 92.2 ±28.5 | 0.104 |
| Albumin (g dl^-1^) | 42.7 (6) | 42.7 (6.5) | 42.4 (5.6) | 0.335 |
| Creatinine (mg dl^-1^) | 0.82 (0.26) | 0.80 (0.21) | 0.88 (0.32) | 0.027 |
| Bilirubin (gm dl^-1^) | 0.50 (0.4) | 0.43 (0.34) | 0.54 (0.46) | <0.001 |
| AST (U l^-1^) | 44.0 (31) | 37.0 (20.3) | 55.5 (56.3) | <0.001 |
| ALT (U l^-1^) | 40.5 (42.8) | 34.5 (28.3) | 49.5 (59.8) | 0.001 |
| GGT (U l^-1^) | 76.0 (88.0) | 57.0 (76.5) | 96.0 (94.8) | <0.001 |
| HIV infection parameters |  |  |  |  |
| CD4 + T-lymphocyte count (cells μl^-1^) | 455 (368) | 514 (426.8) | 353 (362) | 0.002 |
| HIV-RNA <50 copies ml^-1^ | 63% (130/207) | 62% (67/108) | 64% (63/99) | 0.812 |
| HIV-RNA <400 copies ml^-1^ | 76% (157/207) | 73% (79/108) | 79% (78/99) | 0.344 |
| cART | 91% (190/210) | 89% (98/110) | 92% (92/100) | 0.473 |
| PI | 57% (108/190) | 56% (55/98) | 58% (53/92) | 0.836 |
| N(t)RTI | 93% (177/190) | 94% (92/98) | 92% (85/92) | 0.685 |
| NNRTI | 18% (35/190) | 19% (19/98) | 17% (16/92) | 0.723 |
| II/EI | 25% (47/190) | 22% (22/98) | 27% (25/92) | 0.451 |
| HCV infection parameters |  |  |  |  |
| HCV-RNA (log IU ml^-1^) | 5.93 (1.40) | 5.77 (1.51) | 6.08 (1.17) | 0.143 |
| HCV-genotype | 87% (183/210) | 85% (93/110) | 90% (90/100) |  |
| 1 | 56% (104/183) | 59% (55/93) | 54% (49/90) | 0.236 |
| 2 | 2% (3/183) | 2% (2/93) | 1% (1/90) |  |
| 3 | 32% (58/183) | 26% (24/93) | 38% (34/90) |  |
| 4 | 10% (18/183) | 13% (12/93) | 7% (6/90) |  |
| Underwent TE | 54% (114/210) | 52% (57/110) | 57% (57/100) | 0.452 |

**Supplementary table 1.** Comparison of all HIV/HCV-coinfected patients with FIB-4 <1.45 and ≥1.45.

ALT alanine transaminase, AST aspartate transaminase, cART combined antiretroviral therapy, EI entry inhibitors, GGT gamma-glutamyl transpeptidase, HIV human immunodeficiency virus, HCV hepatitis C virus, II integrase inhibitors, IVDU intravenous drug abuse, MSM men who have sex with men, NNRTI non-nucleoside reverse-transcriptase inhibitors, N(t)RTIs nucleos(t)idic reverse transcriptase inhibitors, PI protease inhibitor, TE transient elastography
